# Reconciling differences in impact of molecular subtyping on response to cisplatin-based chemotherapy

**DOI:** 10.1038/s41467-021-24837-8

**Published:** 2021-08-10

**Authors:** Mathieu Roumiguie, Alberto Contreras-Sanz, Gunjan Kumar, Peter C. Black

**Affiliations:** 1 Vancouver Prostate Center, Department of Urologic Sciences, University of British Columbia, Vancouver, Canada; 2 Department of Urology, CHU-Institut Universitaire du Cancer-Oncopôle, Toulouse, France

**Keywords:** Cancer genomics, Bladder cancer

Arising from Ann Taber et al. *Nature Communications* 10.1038/s41467-020-18640-0 (2020)

Predictive biomarkers are urgently required in patients with muscle-invasive bladder cancer to determine which patients will benefit from cisplatin-based chemotherapy (CBC). In their study, Taber et al. used an impressive array of multi-omics platforms to demonstrate that genomic instability predicted favorable response to CBC in bladder cancer, while basal/squamous (Ba/Sq) gene expression predicted a poor response. This manuscript is an important reference article for molecular correlates of response to CBC. However, the results of this study contradict two findings from the literature that are currently being tested in clinical trials in the neoadjuvant CBC (NAC) setting. The first is the finding that response to NAC correlates with the presence of ERCC2 mutations^1–4^ and the second is the finding that basal/squamous tumors appear to benefit more from NAC than other subtypes^5,6^.

Prior studies investigating markers of response to CBC have focused primarily on localized muscle-invasive bladder cancer (MIBC), whereas this study from Taber et al. mixes a predominantly metastatic/locally advanced urothelial carcinoma (mUC, *n* = 110) population with MIBC patients (*n* = 62). CBC is administered with an entirely different intent in each population (curative intent for MIBC vs. prolonging survival for mUC), and the endpoints of response are very different. After NAC for MIBC, response is measured by the absence of residual disease (ypT0N0) or presence of only non-muscle-invasive disease (NMIBC, ypT ≤ 1N0) in the cystectomy specimen^7,8^, while response of mUC to CBC is measured by radiologic imaging, usually based on Response Evaluation Criteria in Solid Tumors (RECIST) criteria. The two endpoints in the different disease states are not necessarily comparable, and conclusions drawn from a mixed population with predominance of mUC may not apply to a MIBC population. A pathologic response to NAC typically results in durable progression-free survival^9^, while an imaging response to CBC in mUC is almost always followed by progression and death, even though the depth of response may correlate to the duration of survival^10^.

In the MIBC setting, a complete transurethral tumor resection at the time of diagnosis can result in an apparent subsequent pathologic response at the time of radical cystectomy after NAC even if NAC has no effect. We have previously demonstrated that this surgical downstaging is most likely with luminal tumors^11^, which also have the best survival, even though it is not clear that they respond to CBC^6^. In the mUC setting, luminal tumors may also have a more indolent course based on a less proliferative tumor biology, but the influence of CBC may be different in this setting where a surgical cure is not part of the equation.

In the study of Taber et al., there appears to be an enrichment for patients who experienced a pathologic response after NAC. In clinical trials, the rate of ypT0 tends to be ~35%, and the ypT ≤ 1 rate ~50%^8,12^, and in real-world practice these numbers are ~10% lower^13^, yet in this study, the pathologic response rate was 63%. Review of the raw supplemental data indicates that 41 of 58 (71%) patients with cT2-4aN0M0 bladder cancer who received NAC and underwent cystectomy had ypT≤1N0 disease. This type of selection bias can influence the performance of predictive biomarkers. Additional confounders are the inclusion of patients who received only 1 or 2 cycles of NAC, 2 patients with cT1N0M0 disease who would not typically receive NAC but ended up with ypT2-4 disease, and one patient with neuroendocrine carcinoma who received carboplatin/etoposide. In the first-line mUC setting it is noteworthy also that 7 patients previously received NAC prior to RC, which would influence clinical outcomes and could influence tumor biology depending on what tissue was used for analysis (which is not indicated in the methods).

We have previously reported that patients with basal tumors benefit most from NAC, and Taber et al. state that these patients have worse prognosis. Importantly, we did not observe a correlation between subtype and pathologic response in our study, and specifically in the Ba/Sq subtype, we did not see a clear correlation between pathologic response and survival. Three key factors need to be considered to align the apparent discrepancy between both studies.

First, it is not clear from the Kaplan–Meier survival curve in Fig. 4c what proportion of the 121 patients in this analysis were being treated with NAC for localized disease. From Fig. 1 the reader can estimate that 12 Ba/Sq patients were treated with NAC and 20 with first-line CPC for advanced disease. Furthermore, the comparison in Fig. 4c is an univariate analysis that is especially missing an adjustment for disease state (MIBC vs. mUC).

Second, the subtype-specific benefit of any treatment can only be assessed when comparing to a similar cohort that underwent no treatment or a different treatment. Our conclusion that patients with basal tumors benefitted most from NAC was based on a comparison to a large cohort treated with RC alone. In the current study, we do not know how tumors of different subtypes would have performed without chemotherapy, so that we cannot conclude if Ba/Sq tumors had more or less benefit.

Finally, Taber et al. compared Ba/Sq to non-Ba/Sq, but the latter is a heterogeneous group, with variable outcomes associated with each subtype. In this dataset, exactly half of non-Ba/Sq tumors are luminal papillary, which generally has the most favorable prognosis in the MIBC setting regardless of NAC.

Although these confounders are considered here primarily in the context of molecular subtypes, some of the same factors apply to analysis of clinical implications of ERCC2 mutation status^14,15^.

Taber et al. provide a comprehensive integrative analysis of molecular features related to response to CBC. While there are differences in the conclusions of this paper compared to prior reports, some of this is likely explained by differences in the clinical context. Expansion of these results and their clinical implementation will require analysis of a greater number of patients with careful consideration of many clinical details of the patient population being investigated. Prospective validation in clinical trials with well-defined inclusion criteria and endpoints will provide the most definitive answers.
